# Evaluation of the Potential Prognostic Value of Tumor Budding in Laryngeal Carcinoma by Conventional and Immunohistochemical Staining

**DOI:** 10.1155/2020/9183671

**Published:** 2020-11-13

**Authors:** Nermine M. Abd Raboh, Ossama M. Mady, Sarah A. Hakim

**Affiliations:** ^1^Department of Pathology, Faculty of Medicine, Ain Shams University, Cairo., Egypt; ^2^Department of Otorhinolaryngology, Faculty of Medicine, Ain Shams University, Cairo., Egypt

## Abstract

**Background:**

Tumor budding is a promising prognostic indicator in several cancers especially in colorectal cancer. However, only few studies have been conducted to assess and validate its prognostic value in laryngeal squamous cell carcinoma; none of which used pancytokeratin immunohistochemistry. In view of the modest results of treatment of laryngeal squamous cell carcinoma, the need of new prognostic indicators becomes of paramount importance. *Aim of the Study*. We aim to evaluate tumor budding in laryngeal squamous cell carcinoma, by haematoxylin and eosin, as well as by pancytokeratin immunohistochemistry. *Material and Methods*. A retrospective study on 118 cases of laryngeal squamous cell carcinoma from archives of Pathology Lab of Ain Shams University Specialized Hospital and Ain Shams University Hospitals from January 2014 to January 2017. The ENT and histopathology reports were reviewed to determine clinicopathologic data of the patients.

**Results:**

Tumor budding shows high statistically significant relations (*p* = 0.0001 for each) with important clinicopathological parameters of laryngeal carcinoma (site, grade, tumor stage, lymph node stage, lymph node extracapsular invasion, and vascular invasion). The extent of tumor budding correlated with overall survival, local recurrence disease free, and distant metastasis disease free (*p* = 0.001 for each). Multivariate analysis showed tumor budding to be an independent prognostic factor affecting progression-free survival. There was a moderate agreement between H&E and IHC by pancytokeratin as regards detection of budding among study cases (kappa = 0.593).

**Conclusions:**

Tumor budding was correlated with poor prognostic clinicopathologic indicators in laryngeal squamous cell carcinoma. It is recommended to use pancytokeratin immunohistochemistry to evaluate tumor budding in laryngeal squamous cell carcinoma especially in confusing cases.

## 1. Introduction

The current treatment results of laryngeal squamous cell carcinoma (SCC) still remain modest, which necessitates adding new prognostic indicators that would aid in tailoring the treatment to suit individual cases in an attempt to improve the treatment outcome [1]. Tumor budding is a histopathological finding identified by counting small clusters of tumor cells less than 5 cells or single cells at the invasive tumor margin [2]. It expresses the tendency of tumor cells to dissociate and detach at the tumor invasive front, which is a primary step for invasive growth followed by metastasis [3]. Tumor budding has been acknowledged as an additional prognostic marker for colorectal carcinoma [4].

However, only few studies have been conducted on laryngeal SCC to assess the prognostic value of tumor budding. Those studies used different techniques for evaluation of tumor budding and were all conducted by haematoxylin and eosin (H&E) stain only. Thus, the current study is aimed at evaluating tumor budding in laryngeal SCC by H&E and by pancytokeratin immunohistochemistry (Ck IHC) and comparing the presence and extent of tumor budding to established clinicopathological parameters.

## 2. Material and Methods

### 2.1. Tissue and Patient Data

The current retrospective study was conducted on 118 cases of laryngeal carcinoma (squamous cell carcinoma). Cases were obtained from the archives of the Pathology Labs of Ain Shams University Specialized Hospital and Ain Shams University Hospitals. Such cases were diagnosed during the period from January 2014 to January 2017. They were obtained by partial or total laryngectomy and neck dissection, followed by adjuvant radiotherapy. The ear, nose, and throat (ENT) reports and histopathology reports were reviewed to determine clinicopathologic data of the patients: age of patients, gender, tumor site, and lymphovascular invasion, as well as lymph node involvement and lymph node extracapsular extension. For each patient, clinical stage at presentation was classified according to AJCC 8th edition [5]. H&E stained full slides were examined to reevaluate the histopathologic diagnosis and grade [6]. The inclusion criteria were as follows: cases that received no neoadjuvant therapy (radio and/or chemotherapy) prior to surgery; cases with enough tissue, with information for all the covariates; and that had performed follow-up and/or received adjuvant therapy at our hospitals. Originally, 134 cases of laryngeal SCC were available during that period; 11 were excluded due to receiving prior neoadjuvant therapy, three cases for insufficient tissue, and two had no follow-up data available because they only had their surgery at our hospitals but received their adjuvant therapy elsewhere from the start. Thus, the total number of cases included in the current study was 118 cases.

ENT reports of the patients were reviewed to determine the following: (a) survival time, which was calculated based on the date of major surgery and the date of last follow-up or death; (b) progression-free survival time, which was calculated based on the date of major surgery or last session of adjuvant postoperative radiotherapy and the date of relapse (local recurrence/distant metastasis) at the last follow-up.

### 2.2. Ethics Statement

All patients who participated in this study signed a written, informed consent before performing the surgery. The study was approved by the Research Ethical Committee at Faculty of Medicine, Ain Shams University.

### 2.3. Histopathological Evaluation

Haematoxylin and eosin (H&E) stained slides of the cases of laryngeal squamous cell carcinoma were examined by two pathologists (the authors: N.M.A. and S.A.H.) to evaluate the presence and grading (extent) of budding using light microscopy at a ×20 objective lens magnification. Any discrepancies were resolved by consensus using a multihead microscope. Budding was considered positive if small clusters of dedifferentiated tumor cells were identified at the invasive margin at any of the sections of the case, with a range of ≥1 cells to ≤5 cells [7, 8].

### 2.4. Immunohistochemical Staining

Four micrometer sections of formalin-fixed and paraffin-embedded samples of the 118 cases of laryngeal squamous cell carcinoma were prepared. For further identifying of tumor budding, immunohistochemical staining was performed using primary antibodies: mouse monoclonal anti-pancytokeratin (catalogue number = NBP2-29429, clone: AE-1/AE-3; Novus Biological Labs, Centennial, Colorado, USA; 0.5-1.0 *μ*g/ml dilution). Avidin-biotin immunoperoxidase complex technique was used according to Hsu et al. [9], by applying the super sensitive detection kit (Biogenex, California, USA). The prepared tissue sections were fixed on poly-L-lysine-coated slides overnight at 37°C. They were deparaffinized and rehydrated through graded alcohol series. Then, the sections were heated in a microwave oven in 10 mM citrate buffer (pH 6.0) for 20 min. After the blocking of endogenous peroxidase and incubation in Protein Block Serum-Free Solution (Dako Cytomation, Glostrup, Denmark) for 20 min, the sections were incubated overnight at 4°C with primary antibodies. Biotinylated anti-mouse immunoglobulin and streptavidin conjugated to horseradish peroxidase were then added. Then, 3,3′-diaminobenzidine as the substrate or chromogen was used to form an insoluble brown product. Finally, the sections were counterstained with haematoxylin and mounted. Sections of human skin were used as positive control for pancytokeratin. Negative control sections were incubated with normal mouse serum instead of the primary antibody.

### 2.5. Interpretation of Immunohistochemical Staining

Immunohistochemical analysis of tumor budding was blindly performed by the two pathologists (the authors: N.M.A. and S.A.H.) without any prior knowledge of the clinicopathological data, following the same criteria used in H&E sections. Any discrepancies were resolved by consensus using a multihead microscope.

### 2.6. Interpretation of Tumor Budding Grading (Extent)

In order to determine the grading (extent) of budding, several sections of each case were examined to choose the largest section of the whole lesion. Budding was classified as follows: mild = budding at ≤1/3 of the entire margin; moderate = budding at 1/3-2/3 of the margin; marked = budding at ≥2/3 of invasive margin [10]. The samples were then classified into two groups: low-grade budding (mild or no tumor budding) and high-grade budding (moderate and marked budding) [11, 12].

### 2.7. Data Management and Analysis

Data was revised, coded, entered on computer, and analyzed using SPSS package version number 20 (SPSS Statistics for Windows, Version 20.0. Armonk, NY: IBM Corp) Qualitative data were expressed as frequencies (*N*) and percentage (%). Chi-square and Fisher's exact test were used to test association between qualitative variables. Fisher's exact test was used to examine the relationship between two qualitative variables when the expected count is less than 5 in more than 20% of cells. *p* value ≤ 0.05 was considered significant. Kappa statistics was used to examine the agreement between tumor budding evaluation by H&E and by pancytokeratin IHC: kappavalues < 0 as indicating no agreement, 0–0.20 as slight, 0.21–0.40 as fair, 0.41–0.60 as moderate, 0.61–0.80 as substantial, and 0.81–1 as almost perfect agreement. Kaplan-Meier curves were used to describe overall and progression-free survival, while log-rank test was used to compare the overall and progression-free survival between the studied groups. Backward multivariate Cox regression analysis was performed for finding the predictors of overall survival and progression-free survival rates.

## 3. Results

**Figure 1:**
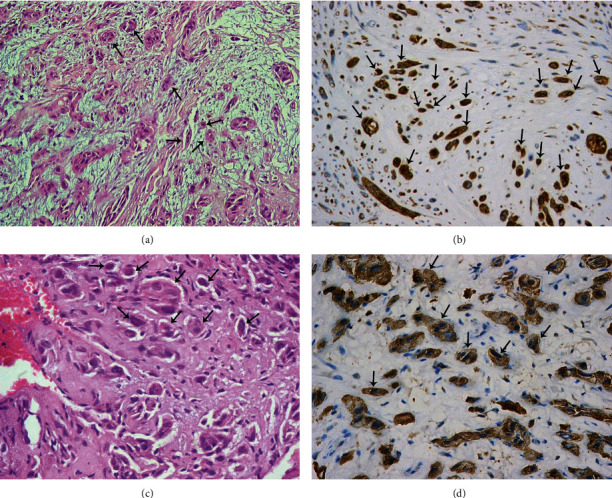
Tumor budding at the invasive front of laryngeal squamous cell carcinoma showing high-intensity tumor budding: (a) by H&E (H&E, ×200), (b) by pancytokeratin (IHC, ×200), (c) by H&E (H&E, ×400), and (d) by pancytokeratin (IHC, ×400). Black arrows are showing examples of the tumor budding.

**Figure 2:**
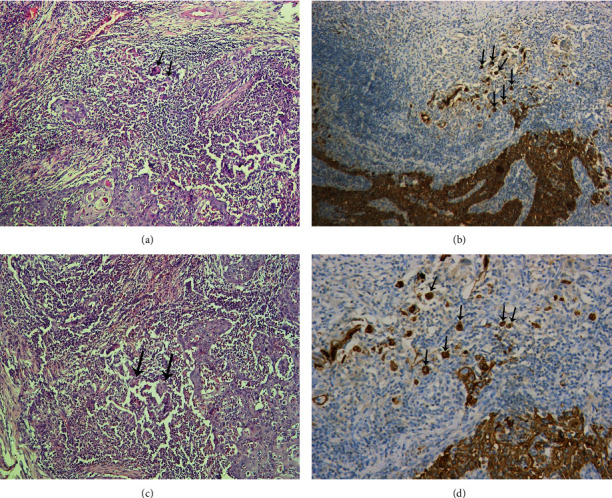
Tumor budding obscured by extensive inflammation and reactive stromal cells: (a) by H&E (H&E, ×100), (b) by pancytokeratin (IHC, ×100), (c) by H&E (H&E, ×100), (d) by pancytokeratin (IHC, ×200). Black arrows are showing examples of the tumor budding.

The study population consists of 118 cases of laryngeal squamous cell carcinoma, 108 (91.5%) males and 10 (8.47%) females. The median age of the patients is 60 years (range, 45-79 years). Fifty-three cases (44.9%) are glottic, 41 cases (34.7%) are supraglottic, 17 cases (14.4%) are transglottic, and 7 cases (5.9%) are subglottic. Pathological stages of the included cases are as follows: stage I (2 cases; 1.7%), stage II (9 cases; 7.6%), stage III (52 cases; 44.1%), and stage IV (55 cases; 46.6%). Fifty-five cases (46.6%) have no lymph node metastasis (N0), 24 cases (20.3%) are N1, and 39 cases (33.1%) are N2 and N3. Thirty-one cases (26.3%) have perinodal invasion. By H&E evaluation, 75 cases (63.6%), 9 cases (7.6%), 8 cases (6.8%), and 26 cases (22%) show no, mild, moderate, and marked budding, respectively. By pancytokeratin IHC evaluation, 37 cases (31.4%), 28 cases (23.7%), 27 cases (22.9%), and 26 cases (22%) show no, mild, moderate, and marked budding, respectively (Figures 1 and 2).

Cases are further classified into high-intensity budding (34 cases, 28.8% by H&E; 53 cases, 44.9% by pancytokeratin IHC) and low-intensity budding (84 cases, 71.1% by H&E; 65 cases, 55.08% by pancytokeratin IHC).

Tumor budding (detected by either H&E or by pancytokeratin IHC) showed highly statistically significant relations (*p* = 0.0001) with important clinicopathological parameters of laryngeal carcinoma. Data showing tumor budding as compared to clinicopathological parameters is summarized in Table 1.

There was a moderate agreement between H&E and IHC as regards detection of budding among study cases (kappa = 0.593), where 75% of cases with no/mild budding by H&E had the same findings by IHC, and 94.1% of cases with moderate/marked budding by H&E had the same findings by IHC (Table 2).

**Figure 3:**
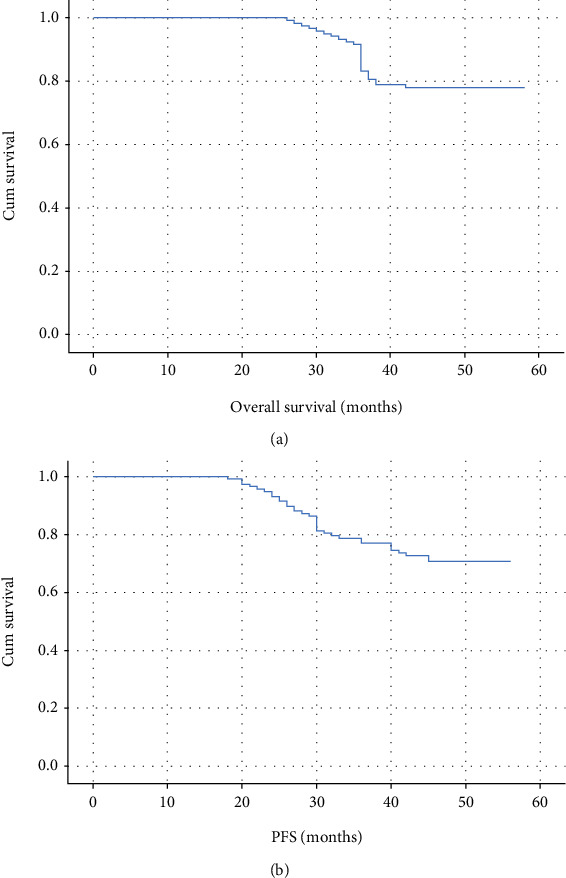
(a) Overall survival among all cases and (b) overall progression-free survival.

The median follow-up of the patients was 48 months (interquartile range ^“^IQR^”^ = 42 − 48 months). The overall 4-year survival (OS) among all cases was 77.9%. The overall progression-free survival (PFS, cases showing neither local recurrence nor distant metastasis) was 70.9% (Figures 3(a) and 3(b)).

**Figure 4:**
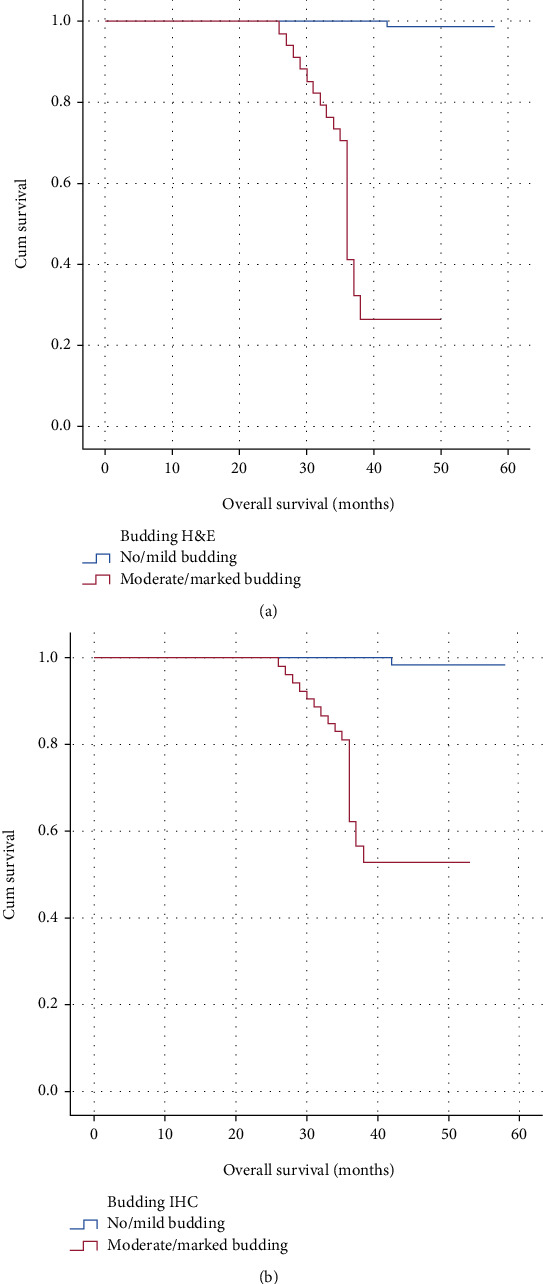
(a) Overall survival in no/mild (low intensity) budding and moderate/marked (high intensity) budding evaluated by H&E; (b) overall survival in no/mild (low intensity) budding and moderate/marked (high intensity) budding evaluated by pancytokeratin IHC.

Low-intensity budding (no/mild budding) and high-intensity budding (moderate/marked budding) had overall survival of 98.8% and 26.5% by H&E (*p* = 0.001) and 98.8% and 52.8% by pancytokeratin IHC (*p* = 0.001), respectively (Figures 4(a) and 4(b)).

**Figure 5:**
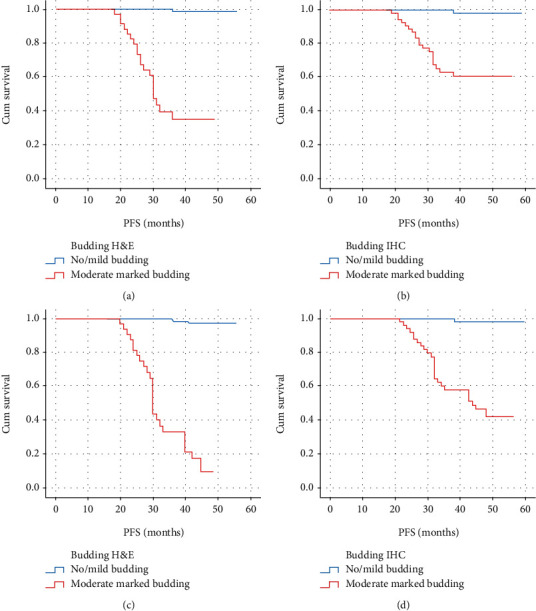
(a) Local recurrence disease-free survival in no/mild (low intensity) budding and moderate/marked (high intensity) budding evaluated by H&E; (b) local recurrence disease-free survival in no/mild (low intensity) budding and moderate/marked (high intensity) budding evaluated by pancytokeratin IHC; (c) distant metastasis-free survival in no/mild (low intensity) budding and moderate/marked (high intensity) budding evaluated by H&E; (d) distant metastasis-free survival in no/mild (low intensity) budding and moderate/marked (high intensity) budding evaluated by pancytokeratin IHC.

For local recurrence-disease free survival, the abovementioned budding scores were 98.8% and 35% by H&E (*p* = 0.001), but 98.5% and 60.4% by pancytokeratin IHC (*p* = 0.001), respectively (Figures 5(a) and 5(b)). For distant metastasis-free survival, the abovementioned budding scores were 97.6% and 8% by H&E (*p* = 0.001), but 98.5% and 43.7% by pancytokeratin IHC (*p* = 0.001), respectively (Figures 5(c) and 5(d)).

After adjustment of all factors (tumor grade, tumor stage, lymph node stage, nodal pericapsular invasion, vascular invasion, and tumor budding) using backward multivariate Cox regression analysis, it was shown that pericapsular invasion and vascular invasion were independent factors affecting overall survival (HR = 33.9, CI = 3.5 − 73.5; HR = 12.7, CI =3.1-51.9, respectively; Table 3).

After adjustment of all factors (tumor grade, tumor stage, lymph node stage, nodal pericapsular invasion, vascular invasion, and tumor budding) using backward multivariate Cox regression analysis, it was shown that lymph node stage, vascular invasion, and tumor budding were independent factors affecting progression-free survival (HR = 11.01, CI = 1.5 − 80.07; HR = 11.8, CI = 3.2 − 42.6; HR = 9.3, CI = 1.8 − 47.6, respectively; Table 4).

Table 5 summarizes the main findings of the current study in comparison to the few previous studies conducted on laryngeal stromal budding.

## 4. Discussion

Laryngeal carcinoma is the second commonest malignancy of head and neck. It includes a heterogenous group of patients with different outcomes, so more appropriate treatment modalities tailored to each case on its own should be offered. This necessitates patient stratification based on other prognostic factors to be added to the traditional, limited WHO grading system [3, 13]. Tumor budding is a microscopic finding detected by light microscopy at the invasive front of tumors. It is defined as the presence of isolated single cancer cells or small clusters of cancer cells (up to 5 cells) [14]. Tumor budding has been reported in several cancers and is considered as a well-established independent prognostic factor in colorectal cancer [4]. Moreover, high reproducibility of tumor budding has been demonstrated in studies conducted on several cancers. Thus, tumor budding could be included in routine histopathological reports of such cancers [15–19]. However, only few studies have been conducted to evaluate the prognostic value of tumor budding in laryngeal carcinoma [20, 21, 22]. None of those studies compared assessing the intensity of tumor budding by pancytokeratin IHC staining to H&E conventional staining. Moreover, the three previous studies did not follow the method used to score the intensity of tumor budding used for colorectal carcinoma. Instead two of those studies [20, 21] followed the technique described by Kanazawa et al. [10], and the third one developed a new technique for estimating tumor budding activity (intensity) [22]. Thus, these two issues (method of scoring and the more appropriate staining technique) require further studies to be conducted to establish solid recommendations for tumor budding in laryngeal cancer in a way resembling the consensus established for colorectal carcinoma. It is worth noting that even this established technique used for assessing the intensity of tumor budding in colorectal carcinoma is still susceptible to further criticism of low interobserver agreement; hence, an automatic counting has been studied [4, 12, 20–22].

Guided by other previous studies that have assessed tumor budding in head and neck squamous cell carcinoma especially those few studies on laryngeal SCC, the method we used for evaluation of tumor budding in the current study demonstrated high statistically significant relations as compared to different clinicopathological parameters including stage, lymphovascular invasion, and lymph node metastasis (*p* = 0.0001 for each). This was in concordance with Ekmekci et al. [20], where tumor budding in their laryngeal carcinoma cases showed statistical significance with lymph node metastases and lymphovascular invasion. Okuyama et al. demonstrated similar correlation between the extent of tumor budding and lymph node metastasis in their gingival SCC cases [23]. Our results also showed partial agreement with their work concerning stage, where they stated that the incidence of budding increased with advanced stage, but their cases showed no statistical significance between budding and stage. Moreover, our study showed total agreement with Boxberg et al. [22], whose work showed a significant correlation between tumor budding and each of tumor stage and lymph node metastases.

Concerning lymph node metastases, although the number, size and level of the invaded lymph nodes were considered important, yet extracapsular spread proved to be of exceedingly paramount prognostic importance [13, 24]. Our current study showed a high statistical significance between tumor budding and extracapsular spread which highlights the probable prognostic value of tumor budding in laryngeal carcinoma. However, our results were unlike Ekmekci et al. [20] and Sarioglu et al. [21], whose cases showed no statistical significance between lymph node extracapsular spread and tumor budding. Among important prognostic factors of laryngeal carcinoma are tumor grade and tumor site [13]. Unlike what was recorded by Ekmekci et al. [20] and Boxberg et al. [22], our cases showed a highly statistically significant relation between tumor budding and each of tumor grade and site (*p* = 0.0001 for each). In this sense, Elseragy et al. [25] proposed a histopathologic grading system that incorporates WHO grading of oral SCC with tumor budding that significantly improved the prognostic power of the conventional WHO grading system. Applying such a prognostically powerful histopathologic grading system in laryngeal SCC might be considered in the future.

The discrepancies between the current study and the few previous studies of tumor budding conducted on laryngeal carcinoma may be attributed to their relatively smaller sample sizes and different means of evaluation of tumor budding extent.

Concerning the staining method of assessing tumor budding, the current study showed only a moderate agreement between staining by H&E and pancytokeratin IHC. In view of unsatisfactory agreement between the two methods of tumor budding evaluation in laryngeal SCC, it is recommended that this evaluation would be performed by pancytokeratin IHC. These results were to a great extent in agreement with Leão et al. [11] whose cases of oral SCC showed fair to moderate agreement between H&E and pancytokeratin IHC. This may be attributed to the fact that observing single tumor cells and small nests can be easily confused with reactive stromal cells by H&E or may be obscured by peritumoral inflammatory infiltrate. However, Wang et al. [17] found almost perfect agreement in evaluation of tumor budding in colorectal carcinoma and so they recommended H&E for reporting tumor budding in colorectal carcinoma. Lugli et al. stated that cost-effectiveness of H&E allows tumor budding evaluation to be applied worldwide, sparing pancytokeratin IHC for challenging cases only [4].

Several studies have demonstrated that tumors associated with high intensity of tumor budding showed a worse prognosis in patients with head and neck SCC [14, 26–28].Tumor budding has been linked to local recurrence, distant metastasis, and lowered survival in many cancers like colorectal carcinomas, pancreatic carcinomas, oesophageal carcinomas, lung carcinomas, anal carcinomas, and even in the few papers conducted on laryngeal carcinomas [8, 29–33].

This goes well with the results of our study, where the overall survival of cases showing no or low-intensity budding and high-intensity budding were 98.8% and 26.5% by H&E (*p* = 0.001), but 98.8% and 52.8% by pancytokeratin IHC (*p* = 0.001), respectively.

As for local recurrence, disease-free survival for no or low-intensity budding and high-intensity budding were 98.8% and 35% by H&E (*p* = 0.001), but 98.5% and 60.4% by pancytokeratin IHC (*p* = 0.001), respectively. Concerning distant metastasis-free survival, budding scores of no or low intensities versus high-intensity budding were 97.6% versus 8% by H&E (*p* = 0.001), but 98.5% versus 43.5% by pancytokeratin IHC (*p* = 0.001), respectively.

These results were in concordance with the studies conducted by Almangush et al. [8, 30], which showed that high-intensity tumor budding significantly correlated with shorter disease-free survival and reduced overall survival in head and neck and oral SCC.

Furthermore, multivariate analysis revealed that tumor budding, lymph node stage, and vascular invasion were independent prognostic factors affecting progression-free survival in our study. This was in agreement with what was demonstrated by Sarioglu et al. [21] in their study that lymph node metastasis and tumor budding were significantly associated with distant metastasis. Moreover, Boxberg et al. showed that their proposed new grading system for laryngeal SCC that incorporates budding activity and cell nest size was significantly associated with overall survival, disease specific, and disease-free survival [22].

Limitations to this study are the small number of patients, unicentricity of the study based in only one location, and using one technique for assessing the intensity of tumor budding. Further studies with wider patients' cohorts, which are comparing the effect of different techniques in assessing the intensity of tumor budding on survival analysis, are required to validate the current results.

## 5. Conclusions

Accumulating evidence has shown that tumor budding could serve as a histopathologic prognostic marker that could even have an influential role in planning the treatment strategy of laryngeal carcinoma patients. However, the best method for its evaluation has not yet been settled upon. Nevertheless, the method used for its evaluation in the current study has shown tumor budding to be an independent prognostic factor for progression-free survival (local recurrence and distant metastasis). Therefore, further studies to establish a consensus for evaluation of tumor budding and for validation of its potential prognostic role in laryngeal carcinoma should be conducted so as to include tumor budding in routine histopathological reports of laryngeal carcinoma.

## Figures and Tables

**Table 1:** Tumor budding in comparison to clinicopathological parameters.

		Budding H&E				*p*	Sig	Budding by IHC				*p*	Sig
		No/mild budding		Moderate/marked budding				No/mild budding		Moderate/marked budding			
		*N*	%	*N*	%			*N*	%	*N*	%		
Site	Glottic	53	63.1%	0	0.0%	0.0001^∗∗^	HS	53	81.5%	0	0.0%	0.0001^∗∗^	HS
	Supraglottic	31	36.9%	10	29.4%			12	18.5%	29	54.7%		
	Transglottic	0	0.0%	17	50.0%			0	0.0%	17	32.1%		
	Subglottic	0	0.0%	7	20.6%			0	0.0%	7	13.2%		

Grade	Grade I	46	54.8%	0	0.0%	0.0001^∗^	HS	46	70.8%	0	0.0%	0.0001^∗^	HS
	Grade II	38	45.2%	7	20.6%			19	29.2%	26	49.1%		
	Grade III	0	0.0%	27	79.4%			0	0.0%	27	50.9%		

Stage	Stage I	2	2.4%	0	0.0%	0.0001^∗∗^	HS	2	3.1%	0	0.0%	0.0001^∗∗^	HS
	Stage II	9	10.7%	0	0.0%			9	13.8%	0	0.0%		
	Stage III	52	61.9%	0	0.0%			52	80.0%	0	0.0%		
	Stage IV	21	25.0%	34	100.0%			2	3.1%	53	100%		

Node stage	N0	55	65.5%	0	0.0%	0.0001^∗^	HS	55	84.6%	0	0.0%	0.0001^∗^	HS
	N1	22	26.2%	2	5.9%			10	15.4%	14	26.4%		
	N2/3	7	8.3%	32	94.1%			0	0.0%	39	73.6%		

Pericapsular invasion	Absent	84	100.0%	3	8.8%	0.0001^∗^	HS	65	100.0%	22	41.5%	0.0001^∗^	HS
	Present	0	0.0%	31	91.2%			0	0.0%	31	58.5%		

Vascular invasion	Absent	84	100.0%	12	35.3%	0.0001^∗^	HS	65	100.0%	31	58.5%	0.0001^∗^	HS
	Present	0	0.0%	22	64.7%			0	0.0%	22	41.5%		

^∗^Chi-square tests; ^∗∗^Fisher's exact test.

**Table 2:** Agreement between H&E and IHC as regards budding detection.

		Budding H&E				Kappa	*p* (sig)
		No/mild budding		Moderate/marked budding			
		*N*	%	*N*	%		
Budding IHC	No/mild budding	63	75.0%	2	5.9%	0.593	0.001
	Moderate/marked budding	21	25.0%	32	94.1%		

^∗^Kappa agreement.

**Table 3:** Backward multivariate Cox regression for evaluating the effect of prognostic histopathologic parameters and tumor budding on overall survival.

	HR	*p*	Sig.	95% confidence interval for HR	
				Lower	Upper
Pericapsular invasion	33.906	0.002	HS	3.510	73.515
Vascular invasion	12.759	0.000	HS	3.133	51.966

^∗^Hazard ratio.

**Table 4:** Backward multivariate Cox regression for evaluating the effect of prognostic histopathologic parameters and tumor budding on progression-free survival.

	HR	*p*	Sig.	95% confidence interval for HR	
				Lower	Upper
Node stage	11.015	0.018	S	1.515	80.076
Vascular invasion	11.847	0.0001	HS	3.289	42.673
Budding^∗^	9.337	0.007	HS	1.829	47.668

^∗^Reference no/mild.

**Table 5:** Summary of the three studies that examined the prognostic value of tumor budding in laryngeal SCC in comparison to the current study.

Points of comparison	Sarioglu et al., 2010 [21]	Ekmekci et al., 2019 [20]	Boxberg et al., 2019 [22]	Current study

Number of cases	64 cases of laryngeal SCC	77 cases of laryngeal SCC	157 cases of laryngeal and hypopharyngeal SCC	118 cases of laryngeal SCC
Study design	Retrospective	Retrospective	Retrospective	Retrospective
Previous neoadjuvant therapy prior to surgery	Not specified	Not specified	Among exclusion criteria	Among exclusion criteria
Staining	H&E only	H&E only	H&E only	H&E+IHC by Ck Only moderate agreement between the two staining methods (*k* = 0.593)
Cutoff for presence or absence of tumor budding	≥1 tumor cell and <5 tumor cells	≥1 tumor cell and <5 tumor cells	≥1 tumor cell and <5 tumor cells	≥1 tumor cell and <5 tumor cells
Intensity (extent) of tumor budding	According to Kanazawa et al., 2008^∗^	According to Kanazawa et al., 2008^∗^	A new technique suggested by the authors^∗∗^	According to Kanazawa et al., 2008^∗^
Survival analysis	O.S., locoregional disease-free, and distant metastasis disease-free survival	Not done	O.S., disease specific, and disease-free survival	O.S. and progression-free survival
Univariate analysis of relation between tumor budding and survival	Tumor budding is significantly associated with distant metastasis-free survival	**____**	Tumor budding activity is significantly associated with O.S., disease specific, and disease-free survival	Tumor budding is significantly associated with O.S. and progression-free survival
Multivariate analysis of prognostic factors and survival	Tumor budding and metastatic lymph nodes are associated with distant metastasis-free survival	**___**	The proposed new grading system (that incorporates tumor budding activity and cell nest size) and metastatic lymph nodes are associated with O.S., disease free, and disease-specific survival	Tumor budding, metastatic lymph nodes and vascular invasion are associated with progression-free survival

^∗^According to Kanazawa et al., 2008. Budding intensity was classified as follows: examined to choose the largest section of the whole lesion. Budding was classified as follows: mild = budding at ≤1/3 of the entire margin; moderate = budding at 1/3-2/3 of the margin; marked = budding at ≥2/3 of invasive margin. ^∗∗^A new technique for evaluating budding activity suggested by Boxberg et al., 2019 in their study: assessed in 10 continuous HPFs in areas showing maximal budding; such that low budding activity was defined as 1 to 14 budding nests and high budding activity as ≥15 budding nests, respectively. SCC: squamous cell carcinoma; IHC: immunohistochemistry; Ck: pancytokeratin.

## Data Availability

All data generated or analyzed during this study is included in this published article.
